# The Impact of Culture Negativity on the Outcomes of Revision Total Knee Arthroplasty for Chronic PJI

**DOI:** 10.3390/microorganisms12071384

**Published:** 2024-07-08

**Authors:** Emily M. Ronan, Garrett Ruff, Itay Ashkenazi, Hayley Raymond, Casey Cardillo, Jordan C. Villa, Ran Schwarzkopf, Vinay K. Aggarwal

**Affiliations:** NYU Langone Health, New York, NY 10003, USA; emily.ronan@nyulangone.org (E.M.R.); itay.ashkenazi@nyulangone.org (I.A.); hayley.raymond@nyulangone.org (H.R.); casey.cardillo@nyulangone.org (C.C.); jordan.villamartinez@nyulangone.org (J.C.V.); vinay.aggarwal@nyulangone.org (V.K.A.)

**Keywords:** rTKA, Periprosthetic joint infection, culture-negative, culture-positive

## Abstract

Culture-positive (CP) and culture-negative (CN) periprosthetic joint infections (PJI) remain a crucial area of research; however, current studies comparing these infections rely on unstandardized outcome reporting tools. Our study aimed to compare the outcomes of two-stage revision of CP and CN PJI using the standardized Musculoskeletal Infection Society (MSIS) outcome reporting tool. We retrospectively reviewed 138 patients who were diagnosed with PJI and indicated for two-stage revision total knee arthroplasty (rTKA). The majority of patients in both CP and CN cohorts achieved infection control without the need for reoperation (54.1% and 62.5%, respectively). There was a significant difference in the overall distribution of MSIS outcomes (*p* = 0.043), with a significantly greater rate of CN patients falling into Tier 1 (infection control without the use of suppressive antibiotics) (52.5% versus 29.6%, *p* = 0.011). There was also a significant difference in the distribution of septic versus aseptic reoperations after 2nd stage (*p* = 0.013), with more CP reoperations being septic and more CN reoperations being aseptic. The duration from first to second stage was significantly shorter in the CN cohort (*p* = 0.002). While overall infection control was similar between cohorts, these data suggest that the outcomes of two-stage rTKA are favorable in cases of CN PJI.

## 1. Introduction

Periprosthetic joint infection (PJI) remains perhaps the gravest complication of TKA and can have numerous catastrophic effects on patient wellbeing [1,2]. Although the incidence of PJI is rare in the United States [3,4], its effects pose a significant reduction in patient quality of life and represent a considerable financial burden to both patients and the healthcare system alike [5,6]. Culture negative (CN) PJI is a rare but relatively prevalent phenomenon that poses a significant clinical challenge to physicians [7]. The prevalence of CN PJI may be attributed to many factors, including the use of antibiotics prior to culture sampling or the delayed transport of such samples [8,9]. Additionally, the use of traditional culture mediums that are limited in their ability to grow atypical organisms, such as those that require a longer incubation period or that are encased in biofilms, are a possible cause of false-negative (CN) results [10]. Regardless of the cause, CN PJI significantly complicates medical decision-making, from choice of antibiotics prescription to surgical treatment options [11].

Research studies regarding outcomes following CN PJI have demonstrated differing results. Some scholars state that CN PJI yields worse outcomes as compared to those of culture positive (CP) PJI patients, arguing that the lack of identification of an infecting organism obscures treatment options and complicates both diagnosis and management of the infection [12,13]. Others, however, have demonstrated that outcomes are more comparable or are even favorable for patients with CN PJI, attributing the negative culture to be indicative of a less severe infection [9,12,14]. The controversy surrounding CP and CN PJI, and what the clinical implications are for each, indicates a need for further research in this area. The ambiguity surrounding CP versus CN PJI outcomes is compounded by the fact that current data comparing these infections rely on unstandardized outcome reporting tools [15]. As such, the Musculoskeletal Infection Society developed an outcome reporting tool to both inform treatment decisions for physicians and provide standardization for researchers evaluating PJI [16].

Our study aimed to compare the outcomes of two-stage revision of CP and CN PJI using this outcome reporting tool. We hypothesized that patients experiencing CN PJI would have unfavorable outcomes (thus, a higher MSIS outcome score) compared to those experiencing CP PJI. The results of this study will provide more standardized evidence of CN PJI outcomes, highlighting the gravity of these infections and informing future clinical decision-making in the context of these infections.

## 2. Methods

### 2.1. Study Design

After obtaining Institutional Review Board (IRB) approval, we retrospectively reviewed a registry of 382 patients with a record of revision total knee arthroplasty (rTKA) from 2011 to 2022 at a single, tertiary academic medical center. Patients were included if they had a PJI diagnosis following TKA.

Both tissue and fluid samples were used as part of the diagnostic process for PJI. Tissue samples were collected from all patients intraoperatively during their 1st stage revision. Several types of tissue sampling were performed, including at least one of the following: periprosthetic membrane, hematoma, bone, synovium, and synovial fluid. Certain patients also underwent preoperative joint aspiration, which was sent for cell count, differential, and a complete microbiological assessment. No swabs were used for sampling.

All tissue and fluid specimens underwent routine gram staining followed by culture on both solid media (agar) and liquid media (thioglycollate broth). Fungal specimens were stained and were plated on solid media. Acid-fast bacilli (AFB) specimens were stained and then inoculated onto both solid and liquid media. Recovered organisms were identified biochemically or in some cases by matrix-assisted laser desorption ionization–time-of-flight (MALDI-TOF) mass spectrometry. In most cases, susceptibility testing was performed using either the VITEK 2 automated system (bioMérieux, Inc., Hazelwood, MO, USA) or the MicroScan system (American MicroScan, Mahwah, NJ, USA). Rarely, the testing was done by manual minimum inhibitory concentration (MIC) determination or by Kirby–Bauer disk diffusion. These methods of sample collection and analysis were consistent throughout the study period (2011–2022).

We defined PJI according to the 2018 International Consensus Meeting (ICM) criteria [17,18,19,20]. As such, major criteria included (a) two positive cultures of the same organism or (b) the presence of a sinus tract with communication to the joint and/or prosthesis. Minor criteria were evaluated using a point system. Minor criteria included an elevated synovial white blood cell count (3 points), presence of intraoperative wound purulence (3 points), presence of positive histology (3 points), elevated synovial polymorphonuclear (PMN) percentage (2 points), presence of one positive culture (2 points), an elevated serum CRP (2 points), and an elevated erythrocyte sedimentation rate (ESR) (1 point). Patients who had at least one major criterion were considered positive for PJI, and those who met minor criteria that amounted to ≥6 points were also considered positive for PJI [19,21].

**Figure 1:**
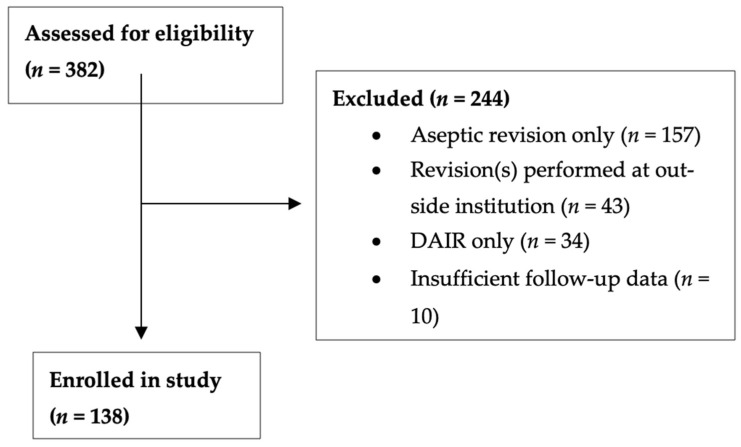
Patients excluded from study.

Patients who met these criteria, who were indicated for two-stage rTKA, who went on to complete at least stage one of the two-stage rTKA at the primary institution, and who had at least one year of follow-up were included in the study. Patients who received debridement, irrigation, and implant retention (DAIR) only as PJI treatment or who underwent aseptic revision only were excluded from the study. Patients whose demographic, surgical, and/or follow up data were incomplete were also excluded. The distribution of patients excluded from the study can be observed in Figure 1.

Cohorts were then defined using the above criteria. Patients were placed in the CN PJI cohort if they met PJI criteria without having a positive culture during the preoperative diagnosis process. Patients who had a negative culture during the preoperative diagnosis process but a positive culture intraoperatively were placed in the CP cohort. All other patients were placed in the CP cohort. All patients in the CP cohort, regardless of the identified species, received the same treatment as those in the CN cohort following PJI diagnosis: two stage revision.

### 2.2. Data Collection

Demographic variables including age, sex, race, smoking status, body mass index (BMI), American Association of Anesthesiologists (ASA) class, and Charlson Comorbidity Index (CCI) score were collected for each patient. For patients in the CP cohort, the species of infecting organism was also recorded. Postoperative data including postoperative antibiotic use, follow-up duration, duration from 1st to 2nd stage, spacer retention status, incidence of unplanned reoperations (including septic and aseptic), and mortality were collected.

Using these outcomes data, patients were then categorized into tiers according to the 2018 MSIS outcomes reporting tool [16]. Tier 1 was defined as infection control without the use of suppressive antibiotics and tier 2 as infection control with the use of suppressive antibiotics. Suppressive antibiotic use was defined as the use of antibiotics for greater than one year postoperatively. As such, if a patient received “prolonged” antibiotic treatment for less than one year postoperatively, they were not considered to be on suppressive antibiotics and were consequently categorized as tier 1. Tier 3 was defined as the need for reoperation or as retention of spacer. Tier 3 was subdivided into 6 categories:

3A: Aseptic revision over one year after the start of PJI treatment;

3B: Septic revision (i.e., DAIR) over one year after the start of PJI treatment (excluding amputation, resection arthroplasty, and arthrodesis);

3C: Aseptic revision less than one year from the start of PJI treatment;

3D: Septic revision less than one year from the start of PJI treatment (excluding amputation, resection arthroplasty, and arthrodesis);

3E: Amputation, resection arthroplasty, or arthrodesis;

3F: Spacer retention.

Tier 4 was defined as all-cause mortality at any time following the initiation of PJI treatment. [16]

### 2.3. Data Analysis

We performed all statistical analysis using SPSS v25 (IBM Corporation, Armonk, NY, USA). All demographic and surgical outcomes data were assessed using chi square analysis and independent sample two-sided t-tests for categorical and continuous variables, respectively. We reported all categorical variables as frequencies with percentages, and we reported all continuous variables as means with standard deviations. Statistical significance was defined as the presence of a *p*-value ≤ 0.05.

## 3. Results

The final study population included 138 patients, with 98 patients in the CP cohort and 40 patients in the CN cohort. The average age of patients in the CP and CN cohorts was 64.01 and 61.25, respectively (*p* = 0.176). The CP cohort was 48.0% male, and the CN cohort was 32.5% male (*p* = 0.097). The average BMI was 31.75 and 33.70 in the CP and CN cohorts, respectively (*p* = 0.145). The majority of patients in the CP and CN cohorts had an ASA score of 3 (58.2% for CP and 52.5% for CN; *p* = 0.524). The average CCI score for the CP cohort was 3.57, while that of the CN cohort was 3.71 (*p* = 0.735). More than half of the patients in the CP cohort had their primary total knee arthroplasty (pTKA) completed at NYU Langone Health, while only 50.0% of patients in the CN cohort had their pTKA completed at NYU Langone Health (*p* = 0.268). Complete results for demographic data can be found in Table 1.

Within the CN cohort, five patients met a major criterion for infection (the presence of sinus tract; 12.5%) and were diagnosed accordingly. The majority of CN patients were diagnosed by meeting minor infection criteria. The complete distribution of major and minor ICM criteria met by patients in the CN cohort can be found in Table 2.

Within the CP cohort, 114 infecting organisms were identified. The most prevalent infecting organism was *Staphylococcus aureus*, with 44 cases comprising 39.8% of all identified organisms. Of these cases, 14 were methicillin resistant. The next most prevalent infecting organism was *Staphylococcus epidermidis*, with 22 cases (19.5% of all identified organisms). Of all CP cases, 13 (13.3%) contained more than one infecting organism (polymicrobial infection). Complete results regarding infecting organisms can be found in Table 3.

The average time to infection (time from primary TKA to PJI diagnosis) within the CP cohort was 46.4 months, while that of the CN cohort was 48.2 months (*p* = 0.877). There was a significant difference in the overall distribution of MSIS outcome scores between patients in the CP and CN cohorts (*p* = 0.043). Significantly fewer patients in the CP cohort had an MSIS tier 1 outcome compared to patients in the CN cohort (29.6% versus 52.5%; *p* = 0.011). There were no significant differences in the number of patients with MSIS tier 2, 3, or 4 outcomes between the two cohorts.

There were no significant differences in the incidence of unplanned reoperations between cohorts (*p* = 0.737). Overall, 87.8% of patients in the CP cohort and 92.5% of those in the CN cohort completed the 2nd stage of their treatment plan (*p* = 0.417). The incidence of unplanned reoperations after 2nd stage in the CP and CN cohorts was 31.4% and 24.3%, respectively (*p* = 0.429). While the rates of unplanned reoperations after 2nd stage were similar between cohorts, the distribution of these reoperations as septic versus aseptic was significantly different, with aseptic revisions comprising 3.5% of all CP and 13.5% of all CN cases that went on to complete their 2nd stage (*p* = 0.013). The average follow-up duration was 41.6 months in the CP cohort and 49.9 months in the CN cohort (*p* = 0.239). The average duration from 1st to 2nd stage was significantly longer in the CP cohort (209.08 days) compared to the CN cohort (139.65 days) (*p* = 0.002). Complete results for surgical outcomes data may be found in Table 4.

## 4. Discussion

Though rare, the incidence of PJI following TKA is a devastating complication that involves extensive treatment which is costly to both the health system and the patients’ health and well-being [22,23]. Among PJI cases in the United States, CN PJI is a rare occurrence in which the diagnosis of infection is made despite failure to grow microorganisms in culture [3,24]. Because a specific microorganism cannot be identified, CN PJI is difficult both to diagnose and to treat [25]. In comparison to those of CP cases, the outcomes of CN PJI are not as well understood [26]. In an effort to compare the outcomes of CP and CN PJI using a standardized model, our study utilized the 2018 MSIS outcome reporting tool to compare the rate of infection control in patients undergoing two-stage rTKA for both CP and CN infections.

Our study demonstrated a significant difference in the distribution of MSIS outcomes between the two cohorts (*p* = 0.043). The majority of CP patients fell into tier 3 (need for reoperation and/or spacer retention), while the majority of CN patients fell into tier 1 (infection control without the use of suppressive antibiotics). There was no significant difference in the number of patients with tier 3 outcomes (*p* = 0.491); however, the percentage of patients with tier 1 outcomes was significantly lower in the CP cohort (29.6%) compared to the CN cohort (52.5%; *p* = 0.011). It is possible that when no specific pathogen was found, physicians were less inclined to administer a non-targeted, wide spectrum suppressive antibiotic therapy, resulting in more CN patients falling into tier 1 compared to CP patients. While the majority of patients in both cohorts achieved infection control (tier 1 or tier 2), these initial results indicate that surgical outcomes following two-stage rTKA are more favorable for CN patients. A similar study by Xu et al. compared 53 CP and 24 CN PJI patients via Kaplan–Meier survival analysis and demonstrated comparable infection control between the two cohorts (*p* = 0.897) [27]. An analogous study by Choi et al. compared the surgical outcomes of 135 CP PJI patients and 40 CN PJI patients, demonstrating an increased success rate of infection control for those in the CN PJI cohort (*p* = 0.006) [28]. However, neither of these studies were specific to TKA, and neither diagnosed PJI according to the 2018 ICM criteria. Most importantly, neither evaluated patient outcomes using the 2018 MSIS outcome reporting tool. To our knowledge, our study is among the first to address all three of these factors. Future studies should employ the MSIS outcome reporting tool in the setting of CP or CN PJI in an effort to continue standardizing outcomes measures for two-stage revisions in these patients.

While there was no significant difference in the number of unplanned reoperations after 2nd stage between cohorts (*p* = 0.429), the distribution of those reoperations as septic versus aseptic was significantly different (*p* = 0.013). While 27.9% of all CP patients who completed 2nd stage underwent septic reoperation, only 10.8% of CN patients underwent septic reoperation. Moreover, the majority of all reoperations in the CP cohort were septic, while the majority of reoperations in the CN cohort were aseptic. While CN infections may often become culture positive after some time [29], it is possible that the cases in our study remained CN and, thus, aseptic after treatment failure, leading to the higher incidence of aseptic reoperations for this cohort. A similar study by Li et al. 2016 compared outcomes of CP and CN patients undergoing two-stage revision and demonstrated that 11.1% of CP patients experienced reinfection while only 7.34% of CN patients experienced reinfection. These results, however, were not statistically significant (*p* = 0.94), and the comparison of reinfection to septic reoperation is not quite analogous. While several studies have shown statistically similar reoperation rates after 2nd stage between CP and CN PJI [30,31], few to our knowledge have elucidated a difference specifically between septic versus aseptic reoperations. The implications of septic reoperation for TKA have been investigated across many studies, the majority of which demonstrating that septic reoperations are associated with reduced patient satisfaction scores, greater cost to patients and the healthcare system, and increased mortality rates [32,33,34]. While these studies are not specific to reoperations following two-stage revision for PJI, they nonetheless indicate the gravity and significant burden of septic reoperations. As our study demonstrates that CP PJI results in a differentially higher ratio of septic reoperations, future studies should investigate the clinical and financial implications of such reoperations so as to inform clinicians in future decision-making.

Within this study, the time between 1st and 2nd stage revisions was significantly longer in the CP cohort than the CN cohort (209.08 days versus 139.65 days, respectively; *p* = 0.002). There are several factors at play in determining the time to 2nd stage revision, including patient comorbidities, laboratory testing, and surgeon preference [12,35]. Moreover, having a positive culture at reimplantation has shown to increase the risk of subsequent reinfection following two-stage revision arthroplasty [36,37,38]. As such, it is possible that having a CP diagnosis influenced surgeons to wait longer periods before reimplantation to ensure that cultures would be negative by the 2nd stage procedure. As previously discussed, while the reoperation rates between cohorts were similar, the distribution of septic versus aseptic revisions was significantly different, with the CP PJI cohort undergoing nearly three times as many septic reoperations as the CN cohort. Thus, our study concurrently demonstrated that CP PJI yields more instances of septic reoperation and a longer time to reimplantation. It would be useful for future studies to explore the relationship between time to reimplantation and septic reoperations in the context of CP and CN PJI. Specifically, studies investigating whether CP PJI and/or time to reimplantation are causative of septic revisions may provide clarity to this area of study.

There are several limitations to this study that should be noted. Firstly, while we attempted to standardize CN PJI diagnosis using the 2018 ICM criteria, there are still several clinical factors that may obscure the parameters of CN PJI diagnosis. In general, infections that are identified as CN may appear as such because of faulty laboratory techniques (such as an inadequate culturing period) or prior antibiotic use before testing [10,39]. While the use of antibiotics prophylactically prior to culture sampling has been debated [40,41], such practice does pose a risk for CN results. Future studies should investigate whether CN PJI patients used prophylactic antibiotics and consider whether this practice has any effect on surgical outcomes. In the future, clinicians may turn to alternative diagnostic methods, such as sonication fluid cultures, to diagnose PJI where a microorganism was not otherwise isolated. Such methodology may have a greater sensitivity in detecting microorganisms, which would mitigate the incidence of CN PJI in the above cases [42].

Another limitation is that our study is retrospective in nature; therefore, our data was prone to errors of medical recording and prone to selection bias. A final limitation of this study is the small sample size of CN cases relative to CP cases. The incidence of PJI following TKA is rare to begin with, and the incidence of CN PJI is rarer still [43]. However, considering the gravity of both CP and CN PJI, more research on the topic of these infections is merited and would greatly benefit surgeons in the long-term.

## 5. Conclusions

Our study compared the outcomes of two-stage revision of CP and CN PJI utilizing the standardized MSIS outcome reporting tool. While the majority of patients in both cohorts achieved infection control without the need for reoperation, the distribution of MSIS outcomes was variable, with a significantly greater number of CN patients falling into tier 1 as compared to the CP cohort. Although treatment of CN PJI may be more challenging for physicians, these initial results demonstrate that CN PJI may in fact have a better prognosis. Future projects should utilize the results of this study to explore treatment options for CN PJI.

## Figures and Tables

**Table 1:** Comparison of demographic variables of between all patients in the CP and CN cohorts.

Demographic Variables			
	CP (*n* = 98)	CN (*n* = 40)	*p*-Value
Age at index revision, years {range}	64.01 {24–89}	61.25 {42–82}	0.176
Sex, *n* (%)			0.097
Male	47 (48.0)	13 (32.5)	
Female	51 (52.0)	27 (67.5)	
Race, *n* (%)			0.351
White	62 (63.3)	19 (47.5)	
Black	16 (16.3)	9 (22.5)	
Asian	2 (2.0)	2 (5.0)	
Other	18 (18.4)	10 (25.0)	
Smoking Status, *n* (%)			0.870
Never	52 (53.1)	23 (57.5)	
Former	39 (39.8)	14 (35.0)	
Current	7 (7.1)	3 (7.5)	
BMI, mean {range}	31.75 {17–54.8}	33.70 {18.2–45.9}	0.145
ASA score, *n* (%)			0.524
1	4 (4.1)	0 (0)	
2	30 (30.6)	15 (37.5)	
3	57 (58.2)	21 (52.5)	
4	6 (6.1)	4 (10.0)	
5	1 (1.0)	0 (0)	
CCI score, mean {range}	3.57 {0–17}	3.71 {0–10}	0.735
Location of pTKA, *n* (%)			0.268
NYU Langone Health	57 (58.2)	20 (50.0)	
other	11 (11.2)	7 (17.5)	
unknown	30 (30.6)	13 (32.5)	

CP, culture-positive; CN, culture-negative; BMI, body mass index; ASA, American Society of Anesthesiologists; CCI, Charlson Comorbidity Index; pTKA, primary total knee arthroplasty.

**Table 2:** Distribution of cases in the CN cohort that were positive for major and/or minor ICM criteria.

ICM Criteria Fulfilled by CN Cohort									
	Major Criteria		Minor Criteria						
	Sinus tract (*n* = 40)	Two positive cultures (*n* = 40)	CRP > 1 mg/dL (*n* = 39)	ESR > 30 mm/h (*n* = 40)	Synovial WBC count 3000 (*n* = 27)	Synovial PMN 80% (*n* = 27)	Intra-op histology (*n* = 28)	Intra-op purulence (*n* = 39)	Positive intra-op culture (*n* = 40)
*n* (%)	5 (12.5)	0 (0)	33 (84.6)	32 (80.0)	19 (70.4)	15 (55.6)	15 (53.6)	18 (46.1)	0 (0)

ICM, International Consensus Meeting; CN, culture negative; CRP, C-reactive protein; ESR, erythrocyte sedimentation rate; WBC, white blood cell; PMN, polymorphonuclear neutrophils; intra-op, intraoperative.

**Table 3:** Infecting organisms identified in all patients within the CP cohort.

Infecting Organism in the CP Cohort		
Organism	*n*	Percentage
*Staphylococcus aureus*	45 (methicillin resistant = 14)	39.5
*Staphylococcus epidermidis*	22	19.3
*Enterococcus faecalis*	7	6.1
*Pseudomonas aeruginosa*	5	4.4
*Staphylococcus lugdunensis*	5	4.4
*Streptococcus agalactiae*	3	2.6
*Klebsiella pneumoniae*	3	2.6
Other:	24	21.0
*Streptococcus orali*	2	1.7
*Streptococcus parasanguinis*	2	1.7
*Cutibacterium acnes*	2	1.7
*Lactobacillus species*	2	1.7
*Streptococcus viridans*	2	1.7
*Candida albicans*	1	0.9
*Staphylococcus hominis*	1	0.9
*Streptococcus mitis*	1	0.9
*Staphylococcus capitis*	1	0.9
*Enterobacter cloacae*	1	0.9
*Peptostreptococcus anaerobius*	1	0.9
*Corynebacterium species*	1	0.9
*Streptococcus angiosus*	1	0.9
*Proteus mirabilis*	1	0.9
*Prevotella species*	1	0.9
*Escherichia coli*	1	0.9
*Acinetobacter calcoaceticus-baumannii complex*	1	0.9
*Actinomyces meyeri*	1	0.9
*Staphylococcus haemolyticus*	1	0.9
Total	114	100

CP, culture positive.

**Table 4:** Comparison of postoperative outcomes between the CP and CN cohorts according to the MSIS outcome reporting tool.

Outcomes of Two-Stage Revision			
	CP (*n* = 98)	CN (*n* = 40)	*p*-Value
MSIS outcome, *n* (%)			0.043
1	29 (29.6)	21 (52.5)	0.011
2	24 (24.5)	4 (10.0)	0.055
3	43 (43.9)	15 (37.5)	0.491
3A	3 (3.1)	1 (2.5)	
3B	3 (3.1)	4 (10.0)	
3C	1 (1.0)	2 (5.0)	
3D	21 (21.4)	3 (7.5)	
3E	7 (7.1)	1 (2.5)	
3F	8 (8.2)	4 (10.0)	
4	2 (2.0)	0 (0)	0.363
Any unplanned reoperations between stages, *n* (%)	17 (17.3)	6 (15.0)	0.737
Completed 2nd stage, *n* (%)	86 (87.8)	37 (92.5)	0.417
Any unplanned reoperations after 2nd stage, *n* (%)	27/86 (31.4)	9/37 (24.3)	0.429
Aseptic	3/86 (3.5)	5/37 (13.5)	0.013
Septic	24/86 (27.9)	4/37 (10.8)	
Follow-up duration, months {range}	41.6 {1.7–124}	49.9 {0–150.6}	0.239
Duration from 1st to 2nd stage, days {range}	209.08 {64–854}	139.65 {54–444}	0.002
If >365 days, reason for delay:			
Unplanned reoperations between stages, *n* (%)	2 (18.2)	1 (100)	
Contralateral joint required surgery between stages, *n* (%)	2 (18.2)	0 (0)	
Patient postponed, *n* (%)	3 (27.3)	0 (0)	
Other, *n* (%)	4 (36.4)	0 (0)	
Total, *n*	11	1	

CP, culture-positive; CN, culture-negative; MSIS, Musculoskeletal Infection Society.

## Data Availability

The raw data supporting the conclusions of this article will be made available by the authors on request.
